# Proline pre-conditioning of Jurkat cells improves recovery after cryopreservation

**DOI:** 10.1039/d3md00274h

**Published:** 2023-07-27

**Authors:** Alex Murray, Peter Kilbride, Matthew I. Gibson

**Affiliations:** a Department of Chemistry, University of Warwick Gibbet Hill Road CV4 7AL Coventry UK m.i.gibson@warwick.ac.uk; b Division of Biomedical Sciences, Warwick Medical School, University of Warwick Gibbet Hill Road CV4 7AL Coventry UK; c Asymptote, Cytiva Chivers Way Cambridge CB24 9BZ UK

## Abstract

Cell therapies such as allogenic CAR T-cell therapy, natural killer cell therapy and stem cell transplants must be cryopreserved for transport and storage. This is typically achieved by addition of dimethyl sulfoxide (DMSO) but the cryoprotectant does not result in 100% cell recovery. New additives or technologies to improve their cryopreservation could have major impact for these emerging therapies. l-Proline is an amino acid osmolyte produced as a cryoprotectant by several organisms such as the codling moth *Cydia pomonella* and the larvae of the fly *Chymomyza costata*, and has been found to modulate post-thaw outcomes for several cell lines but has not been studied with Jurkat cells, a T lymphocyte cell line. Here we investigate the effectiveness of l-proline compared to d-proline and l-alanine for the cryopreservation of Jurkat cells. It is shown that 24-hour pre-freezing incubation of Jurkat cells with 200 mM l-proline resulted in a modest increase in cell recovery post-thaw at high cell density, but a larger increase in recovery was observed at the lower cell densities. l-Alanine was as effective as l-proline at lower cell densities, and addition of l-proline to the cryopreservation media (without incubation) had no benefit. The pre-freeze incubation with l-proline led to significant reductions in cell proliferation supporting an intracellular, biochemical, mechanism of action which was shown to be cell-density dependent. Controls with d-proline were found to reduce post-thaw recovery attributed to osmotic stress as d-proline cannot enter the cells. Preliminary analysis of apoptosis/necrosis profiles by flow cytometry indicated that inhibition of apoptosis is not the primary mode of action. Overall, this supports the use of l-proline pre-conditioning to improve T-cell post-thaw recovery without needing any changes to cryopreservation solutions nor methods and hence is simple to implement.

## Abstract 2

T-cells incubated with proline have superior recovery following cryopreservation, increasing cell yields without changing the actual cryopreservation process.

## Graphical abstract

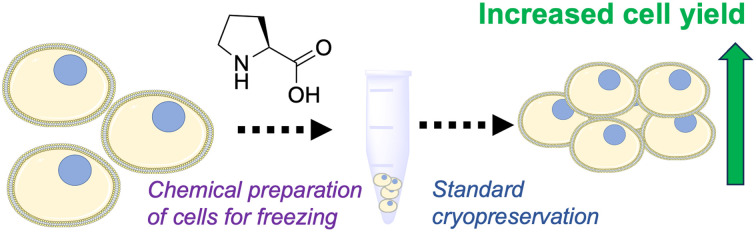

## Introduction

Cell-based therapies are typically cryopreserved after their manufacture for transport and delivery to patients. As an example: chimeric antigen receptor (CAR) T-cells are a cancer treatment consisting of autologous T-cells which have been harvested from a patient, and then genetically modified to express a CAR against a target antigen known to be expressed by the tumour cells, but not by healthy host tissue. These cells (and other cell-based therapies) are expanded before being transfused back into the patient.^1–3^ During these processes cells may be cryopreserved twice, once for transport from the patient to a centralised lab for modification and expansion, and a second time when transporting cells from the lab back to the patient.^4^ As with any cryopreservation process, the thawed cells can show reduced viability and not 100% are recovered.^5^ It should be noted that the effectiveness of CAR T-cell therapies post-thaw is maintained^5,6^ and that reduced recovery compared to fresh does not necessarily mean a therapy is not functional. There are examples of reduced cellular function post-thaw: natural killer (NK) cell therapies were less effective in mice than fresh NK cells, with frozen NK cells having lower viability and homing to different areas of the body.^7^ In melanoma patients, cryopreserved NK cells exhibited lower viability and activity compared to fresh.^8^ In contrast peripheral blood stem cell grafts post-thaw matched the performance of fresh cells.^9^ It is likely that future cell therapies will face similar challenges, and while advances in technology and logistics along with non-frozen cell storage solutions such as hydrogels^10^ may emerge, cryopreservation will remain essential.

The most common cryoprotective agent (CPA) for mammalian cells is DMSO, which can replace intracellular water and reduce ice formation. It is widely used, typically between 5 and 10 wt% (with higher concentrations for vitrification, not discussed here^11^) but can show toxic effects upon transfusion^12,13^ which is mitigated by clinical success of the frozen cells.

During the development of new cryoprotectants, extremophiles in nature provide a source of new targets and strategies. For example, ice-binding proteins (and polysaccharides^14^) have been explored to modulate ice formation and growth,^15–17^ and synthetic mimics have emerged.^18–22^ Trehalose, a non-reducing disaccharide produced by extremophiles,^23^ has also found application but is non-cell penetrating.^24,25^ Many cold or freeze tolerant organisms produce the amino acid l-proline as an osmoprotectant. Only the l-isomer is found in the proteins of mammals, while d-isomers in mammals are typically from bacterial origin.^26,27^ The wood frog *Rana sylvatica* accumulates proline along with several other low molecular weight CPAs before freezing over winter.^28^ The overwintering larvae of the codling moth *Cydia pomonella*, accumulates l-proline and trehalose to survive sub-zero tempeartures,^29^ and the larvae of the drosophilid fly *Chymomyza costata* use proline (in part) to survive exposure to liquid nitrogen temperatures (−196 °C or lower).^30^l-Proline is also used to regulate osmolarity including in fish^31^ and plants.^32^

Unlike trehalose, l-proline is membrane permeable as it can enter the cell through Na^+^ dependent transporters.^33^l-Proline has been supplemented into many cryopreservation solutions including for ovine red blood cells (RBCs), human epithelial cells, mouse fibroblasts, human smooth muscle cells, human spermatozoa and erythrocytes.^34–36^ The mechanism of how l-proline protects is under investigation. It has biophysical effects including reducing intracellular ice formation and may prevent damage through freeze induced dehydration/freeze induced concentration of solutes. However, there is also evidence of distinct biochemical effects of l-proline; pre-conditioning^37^ cells with l-proline prior to cryopreservation has been observed to increase post-thaw cell yields, and feeding l-proline to the larvae of *Drosophila melanogaster* enabled survival at −5 °C.^38^ Bailey *et al.* showed that pre-incubation with l-proline improved neuroblastoma cell monolayer recovery post-thaw, including the observation that the pre-incubation of cells with l-proline transiently reduced cell growth rate.^39^ Similarly, pre-incubation of A549 cell monolayers or spheroids with l-proline increased post-thaw yields, restricted pre-freeze cell proliferation and whole-cell proteomics indicated a broad upregulation of many proteins.^40,41^ P493 (B lymphoma) cells incubated in proline result in the l-proline biosynthesis pathway being inhibited, impacting on the glycolytic pathway which may explain the inhibitor effect, in this particular cell.^42^ These observations support a hypothesis that l-proline is not only acting as an osmolyte, but when exposed to cells for sufficient time periods and concentrations can induce a pre-conditioning effect. There is evidence l-proline can act as reactive oxygen species (ROS) scavenger,^43^ and act as a chaperon, preventing the aggregation of damaged and misfolded proteins.^44^ Another factor (relevant for the results in this study) is cell density. Cell density is an important variable in cryopreservation, but this may not be controlled for in clinical settings where the final cell number (not number initially cryopreserved) is the key measurement. Cell density has been shown to affect the proliferation of Jurkat cells, with growth rate reaching its maximum at an optimal density,^45^ and cell density will impact the total frozen fraction of the cryopreservation solution.^46^

Considering the above, the aim of this study was to evaluate if l-proline could be used to pre-condition Jurkat cells for cryopreservation to improve post-thaw yields and/or viability. Jurkat cells are a useful model for (engineered) T-lymphocytes used in cell-based therapies.^47^ We demonstrate that pre-incubation of Jurkat cells with 200 mM l-proline reduces their proliferation rate, but leads to a subsequent increase in the post-thaw total cell recovery, in a cell-density dependent manner. Direct addition into the cryopreservation medium had no impact. d-Proline had no beneficial effect although another amino acid l-alanine is confirmed to have some benefit too. Reduced metabolic activity, and inhibition of apoptosis were ruled out as protective mechanisms. These results show l-proline is a simple additive to improve T-cell recovery post-cryopreservation.

## Materials and methods

l-Proline (purity >98.5%), d-proline (purity ≥99%) and l-alanine (purity >98.5%) were obtained from Sigma-Aldrich. Solutions were made by dissolving additives in culture media (details of culture media below). All amino acid solutions were sterile filtered with 0.2 μm syringe filters before application to cells. Filters were obtained from Fisher.

### Cell culture

E6.1 Jurkat cells were from the European Collection of Authenticated Cell Cultures (ECACC). The cell line was maintained in T175 flasks (Greiner Bio-One Ltd), and incubated at 37 °C in a humid atmosphere with 5% CO_2_. Complete culture media was Advanced RPMI 1640 supplemented with 1% antibiotic–antimycotic (both Thermo Fisher) and 10% non-USA origin fetal bovine serum (Merck). Base Advanced RPMI 1640 media contained 0.17 mM l-proline and 0.10 mM l-alanine. Cells were passaged every 4 days to maintain a cell density not exceeding 1.5 × 10^6^ cells per mL.

### Cryopreservation, recovery, and viability assays

Cells were taken from the main cell line when its cell density reached approximately 1.0 × 10^6^ cells per mL, and were pre-incubated for 24 hours with an amino acid, or fresh media as the control. Cells were then incubated at room temperature with fresh media and 5% DMSO for 10 minutes. After incubation with DMSO, cells were cooled at −1 °C min^−1^ to −80 °C using a cryovial cooler (Coolcell LX, Corning). Cells were kept at −80 °C for at least 24 hours. Vials were warmed in a 37 °C water bath for 5 minutes, resuspended in fresh media and plated onto a sterile 12 well tissue culture plate (Corning). Cells were incubated at 37 °C in a humid atmosphere with 5% CO_2_ until counting. Centrifugation for the resuspension steps was conducted at 825 × *g* in a Spectrafuge 6C for volumes above 1 mL and at 3000 × *g* in a VWR Micro Star 17 for volumes 1 mL or lower. Cells were counted using a Countess automated cell counter with Countess cell counting chamber slides (both Thermo Fisher). Percentage recovery was defined as 
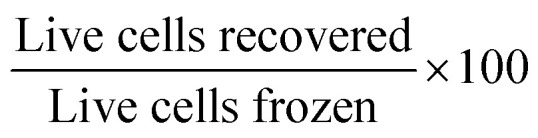
 and viability was defined as 
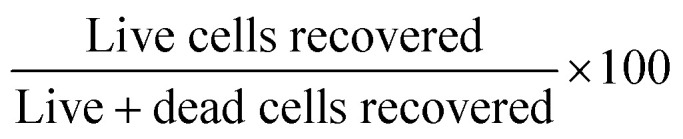
. Growth was defined as 
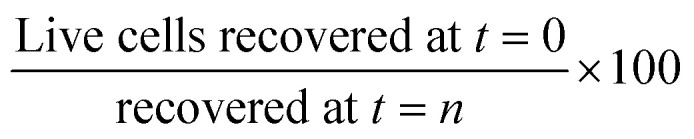
 live and dead cells were distinguished using the trypan blue exclusion method, whereby live cells exclude the dye while dead cells were stained. This was detected with the automated cell counter. Alternatively, dead cells could be detected by microscopy as they are stained blue. The stock trypan blue was obtained from Merck and diluted two-fold with DPBS (Sigma-Aldrich).

### Physical and analytical methods

#### Statistics

Statistical analysis was performed using Prism 9 software (GraphPad). Statistical significance was determined by ordinary one-way ANOVA using Dunnett's test for multiple comparisons, unless only two conditions were compared, in which case a *t*-test was used. *P* values below 0.05 were considered statistically significant.

#### Flow cytometry

Immediately after thawing, cells were labelled with FITC-annexin V, and PI, and the resulting fluorescence was detected by flow cytometry. Fluorescence was first measured 1 hour post-thaw, and the process was repeated for measurements at 4, 8, and 24 hours post-thaw. Flow cytometry was performed using a BD Accuri C6 flow cytometer. 10 000 events were recorded per sample using a flow rate of 14 μL per min^−1^. Data was analysed using FlowJo software. FITC was excited using a 488 nm laser and detected *via* a 533/30 nm bandpass filter. PI was excited using a 488 nm laser and detected using a 585/40 nm bandpass filter. FITC fluorescence spillover into the PI channel was colour compensated by subtracting 14.47% of FITC from PI.

#### Resazurin assay

The previously reported method was adapted for use here.^48^ Resazurin solution was prepared by dissolving one 0.25 g resazurin tablet (Scientific Laboratory Supplies) in 30 mL fresh complete media containing no additives, for a resazurin concentration of 0.0083. Cells were resuspended in resazurin solution and incubated at 37 °C for either 3 or 4 hours. After incubation, the plate was excited with light using a 530/25 mm bandpass filter and fluorescence was detected with a 590/35 nm bandpass filter using a Biotech Synergy HT microplate reader and reported as a % of controls.

## Results and discussion

**Fig. 1:**
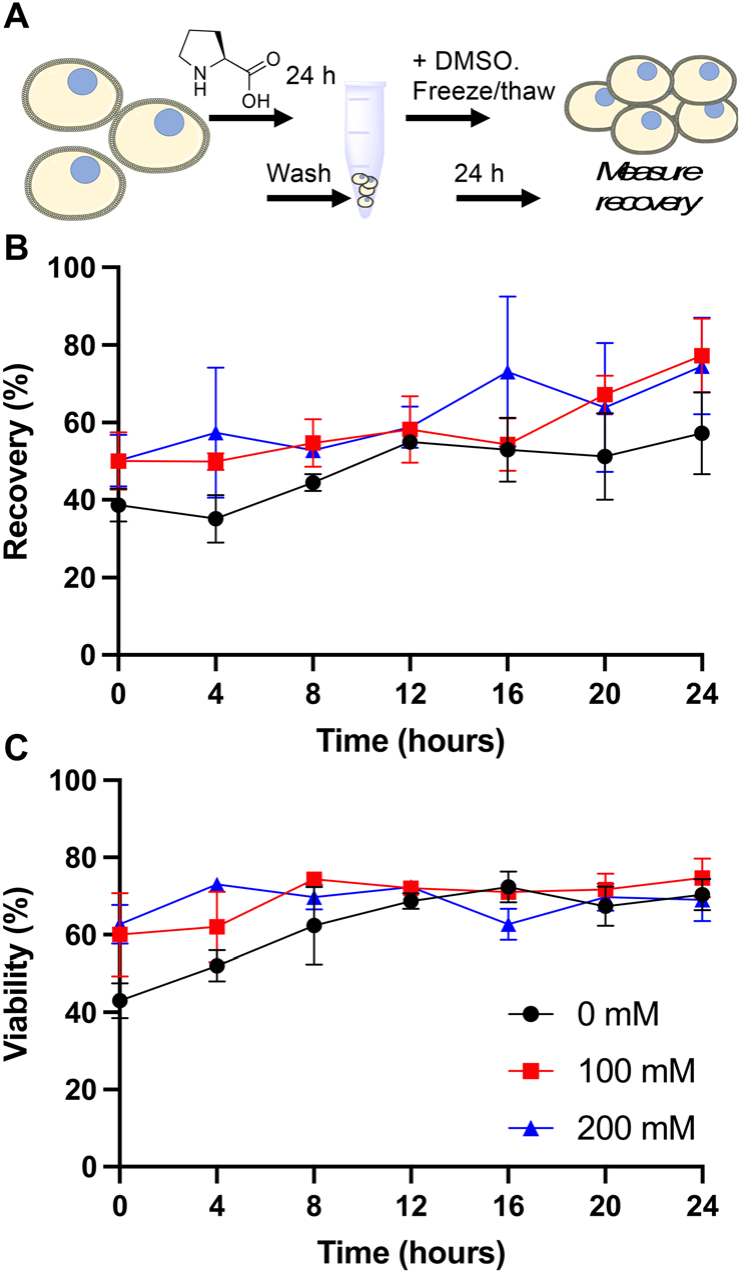
l-Proline pre-incubation impact on Jurkat cell cryopreservation. A) Schematic of cryopreservation process; B) recovery and C) viability of Jurkat cells post-thaw. Prior to freezing, cells were incubated for 24 hours with media containing 0, 100 or 200 mM added l-proline. This media was removed and replaced with fresh media, followed by cryopreservation in 5% DMSO at −1 °C min^−1^ to −80 °C. After 24 hours, cells were at warmed 37 °C. Data represents the mean ± SD of three independent experiments.

Our primary aim was to evaluate if the protective osmolyte l-proline, which has been demonstrated to improve post-thaw recoveries of some adherent cell lines,^39,40^ could improve the post-thaw outcomes of Jurkat cells and/or reduce the DMSO required for cryopreservation. As an initial screen for function, Jurkat cells were cryopreserved in 5% DMSO at a density of 1 × 10^6^ cells per mL which is in the typical range for these cells.^49^ Prior to freezing, the cells were incubated at 37 °C in media supplemented with 0, 100 or 200 mM of l-proline for 24 hours, the media was removed (by centrifugation) and replaced with fresh media prior to cryopreservation to ensure there was no additional proline in the extracellular media (which is studied below). Hence, there was no additional proline in the cryopreservation nor thawing media, although proline would have remained in the intracellular space. Post-thaw, cells were allowed to recover for 24 hours to ensure apoptosis could set in, and to avoid over-estimation of recovery associated with short post-thaw incubations.^50,51^ Recovery and viability was recorded every 4 hours during this period, determined by trypan blue exclusion, Fig. 1.

**Fig. 2:**
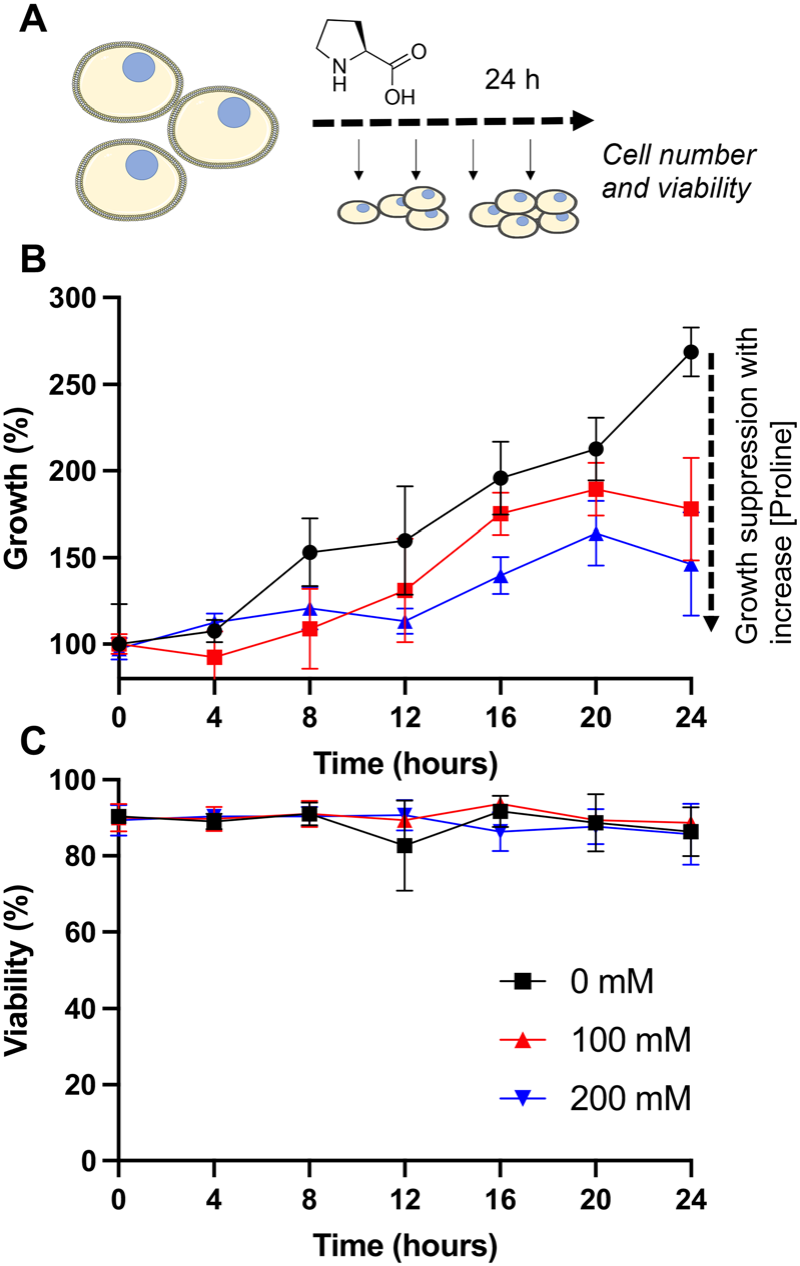
Impact of l-proline supplementation into culture media without cryopreservation. A) Schematic of experiment; B) growth and C) viability of Jurkat cells during 24 hours incubation with media containing 0, 100 or 200 mM added l-proline. Cells were seeded at 1 × 10^6^ mL^−1^. The media already contained 0.17 mM l-proline and 0.10 mM l-alanine and was supplemented with 0 mM, 100 mM, or 200 mM l-proline. Cell count taken every 4 hours. Data represents the mean ± SD of three independent experiments.

The data in Fig. 1B revealed a small increase in post-thaw cell number (relative to pre-cryopreservation) when l-proline was supplemented into the media, with the largest differences after 24 hours with a ∼15% increase in cell yield (39% *vs.* 50% *vs.* 50% and 57% *vs.* 77% *vs.* 75% at the 0 and 24 hour timepoints respectively for 0 mM, 100 mM and 200 mM respectively). Not all these differences were statistically significant at *P* < 0.05, however. The cell viability remained consistent across all conditions. In previous work using A549 (adherent) cells, l-proline pre-incubation increased post-thaw cell yields by >20% in some cases, and also when used in combination with trehalose for Neuro-2a cells,^39^ but from a lower starting point due to the acknowledged challenges of monolayer cryopreservation.^40,52^ During this earlier work, it was observed that the l-proline pre-incubation reduces the overall growth rate of the A549 cells, which may suggest a protective mechanism associated with metabolic pre-conditioning/growth rate suppression.^37^ To probe the effect of l-proline on Jurkat proliferation, cells were cultured in the presence of 0 mM, 100 mM, or 200 mM l-proline, and growth/viability monitored for 24 hours, Fig. 2.

Addition of 100 or 200 mM l-proline lead to a clear and statistically significant decrease in growth rate, nearly halving compared to the media alone (Fig. 2B), highlighting the impact it has on cellular growth, whilst having no impact on the overall cell viability (Fig. 2C), and is discussed later in this manuscript. The effect was dose dependent with 200 mM l-proline inhibiting growth more than 100 mM proline (34% *vs.* 46% reduction in growth after 24 hours compared to control respectively), although the difference between the two l-proline concentrations tested is not statistically significant. This data also shows why l-proline should not be in the thawing or freezing media, to ensure the cells proliferate post-thaw.

To probe the cryoprotective mechanism of l-proline, we compared it to two other amino acids: d-proline and l-alanine. In nature, d-proline is only found in a very small number of proteins and in mammals, most d-amino acids are of bacterial origin.^26^ If d-proline had the same cryoprotective effect as l-proline, it would provide evidence that proline's cryoprotective effect is biophysical rather than biochemical, since cells cannot interact with d-proline on a metabolic level to the extent that they can with l-proline. l-Alanine shares some of the physical mechanisms of cryoprotection with l-proline by virtue of being a water soluble small molecule, but may have different biochemical/metabolic properties inside the cell. For example, l-proline is an epigenetic modulator, chemical chaperone, and a modulator of signalling pathways involved in cell stress.^53^l-Alanine is required for T cell protein synthesis and activation,^54^ and acts as an energy sensor and activates the AMPK pathway. It is important to note that the amino acids are removed from the extracellular space prior to cryopreservation in this present work, to ensure that any effects are limited to the intracellular space.

**Fig. 3:**
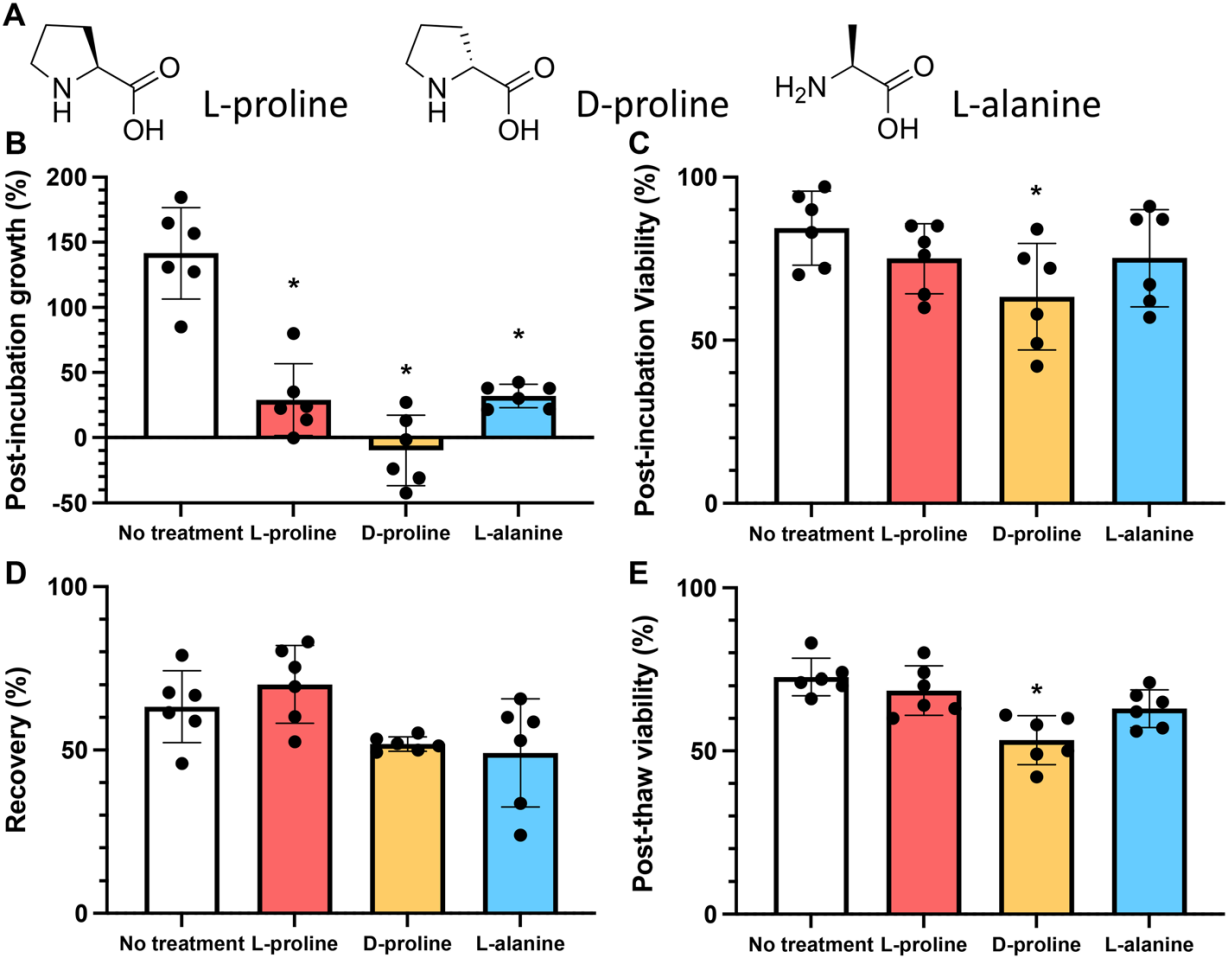
Comparison of amino acids effect on Jurkat cell growth and viability. A) Chemical structures of amino acids; B) growth and C) viability of Jurkat cells after 24 hours incubation with 200 mM of each additive. Cells seeded at a density of 1 × 10^6^ mL^−1^; D) recovery and E) viability of Jurkat cells post-thaw after 24 hours incubation with 200 mM additives and resuspension in fresh non-additive media. The total number of cells frozen in each experiment was identical 1 × 10^6^ mL^−1^. Cryopreservation was undertaken in 5% DMSO at −1 °C min^−1^ to −80 °C and thawed at 37 °C and data collected 24 hours post-thaw. Data represents the mean ± SD of at least 6 independent experiments (*P < 0.05 from untreated control).

As above, the growth rate of cells in the presence of 200 mM of l-proline, d-proline, and l-alanine was measured for 24 hours. All conditions reduced the growth rate of the cells (by 79%, 107% and 72% respectively) (these values were statistically significant). Fig. 3B. However, only d-proline caused a statistically significant reduction (25%) in the proportion of viable cells (Fig. 3C). Cells cryopreserved with l-proline exhibited slightly higher recovery than the untreated control (63% *vs.* 70%), whereas cells cryopreserved with d-proline and l-alanine exhibited lower recovery (52% and 49% respectively) (Fig. 3D). These differences were not statistically significant. Again d-proline lead to a reduction in viability suggesting it is not tolerated by the cells (explored more below). This agrees with observations on A549 (ref. 40) and Neuro-2a^39^ adherent cell lines that proline slows their growth, and also supports increasing post-thaw yields.

**Fig. 4:**
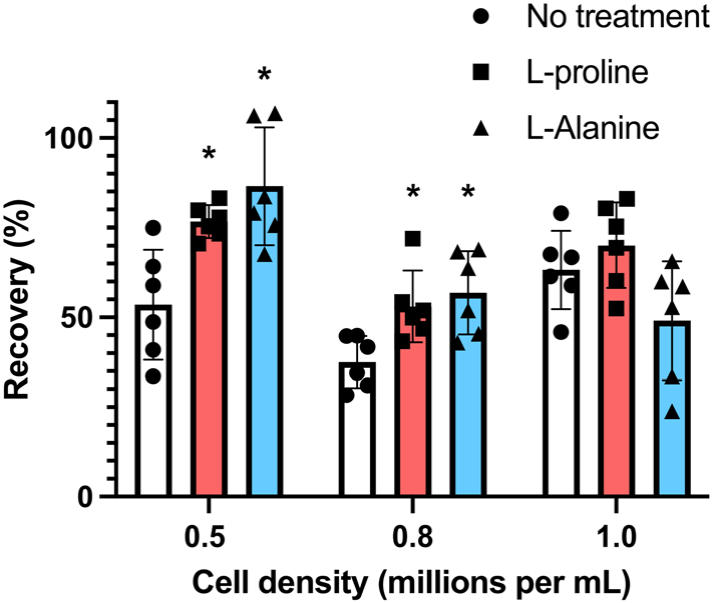
Recovery of Jurkat cells following pre-incubation with 200 mM of indicated amino acids at different cell densities. Cryopreservation was undertaken in 5% DMSO at −1 °C min^−1^ to −80 °C and thawed at 37 °C water bath, and data collected after 24 hours. Prior to freezing, cells were incubated for 24 hours with 200 mM l-proline or 200 mM l-alanine. These additives were removed and replaced with fresh media pre-freeze (*P < 0.05 from untreated control at the same density).

Many cell types are sensitive to cell density, exhibiting density dependent growth rates^55^ and recoveries after cryopreservation.^49^ To explore the effectiveness of the additives at different cell densities, we incubated the cells at densities of 0.5 × 10^6^ mL^−1^ and 0.8 × 10^6^ mL^−1^ (the earlier data from experiments at 1 × 10^6^ mL^−1^ is included here for comparison). Cells were first incubated for 24 hours with the indicated concentrations of amino acids, and after adjustment to 1 × 10^6^ cells per mL (to ensure all were frozen under identical conditions) in fresh media, they were cryopreserved in 5% DMSO. d-Proline was omitted due to the above cytotoxic effects. Fig. 4 shows post-thaw recovery data, showing that cells incubated with l-proline or l-alanine at lower density (0.5 × 10^6^ mL^−1^) exhibited the highest recovery increase (77% and 83% respectively), compared to 53% for the control l-proline has a modest cryoprotective effect at the highest cell density (1 × 10^6^ mL^−1^) and l-alanine has none at all. Both were effective at intermediate densities (0.8 × 10^6^ mL^−1^). Differences between the untreated control, and the l-proline and l-alanine groups at 0.5 × 10^6^ and 0.8 × 10^6^ were statistically significant. The difference between the 0.8 × 10^6^ and 1.0 × 10^6^ treated groups was not statistically significant, however, the difference between the 0.8 × 10^6^ and 1.0 × 10^6^ untreated groups was statistically significant (Fig. 4).

**Fig. 5:**
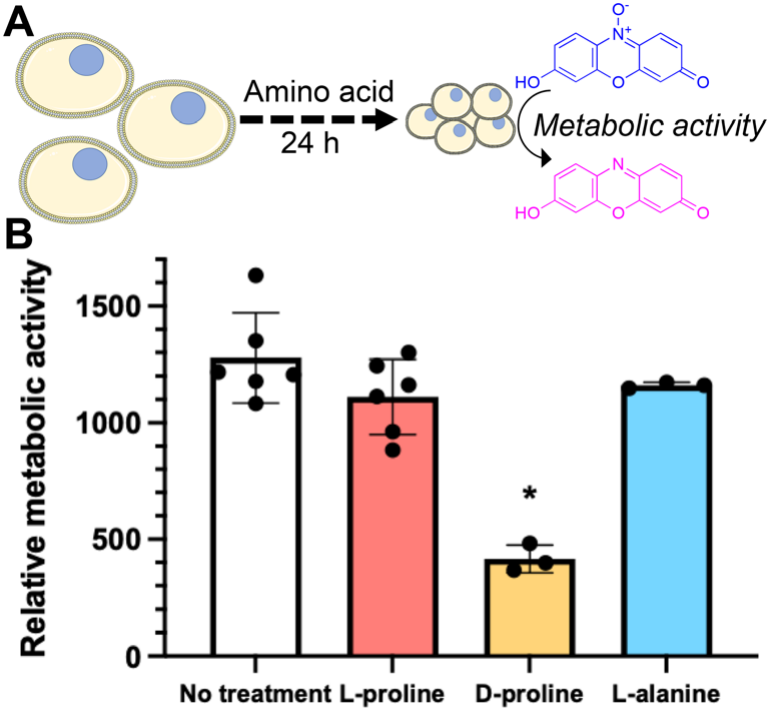
Impact of amino acid incubation on metabolic activity of Jurkat cells. A) Schematic of experiment; B) metabolic activity after 24 hours of incubation. Cells were incubated with media containing 200 mM of each additive for 24 hours and metabolic activity determined by a resazurin reduction assay. Data represents the mean ± SD of at least three independent experiments (*P < 0.05 from no treatment).

Some of l-proline's physical (rather than biochemical) cryoprotective effects including water binding and glassy pocket formation apply to the extracellular space. Control experiments where l-proline was added into the cryopreservation media immediately before freezing (without pre-incubation) led to no increase in recovery, and hence confirming the biochemical impact of this additive to Jurkat cryopreservation. To further probe the role of l-proline we investigated the effect of the additives on cellular metabolism. Cells were incubated for 24 hours in the presence of 200 mM of l-proline, d-proline, l-alanine, or no additive. After this time the concentration of cells was corrected to 1 × 10^6^ cells per mL and the metabolic activity was assessed using a resazurin assay. It was found that only cells incubated with d-proline showed significantly reduced metabolic activity which is consistent with the toxicity observed in the earlier experiment, Fig. 5B. Cells incubated with l-proline and l-alanine also showed reduced metabolic activity, but this was not statistically significant from the control. This suggests that decreasing metabolism alone is probably not responsible for the cryoprotective effect, rather than preventing cell growth, but cannot be ruled out as a contributor.

**Fig. 6:**
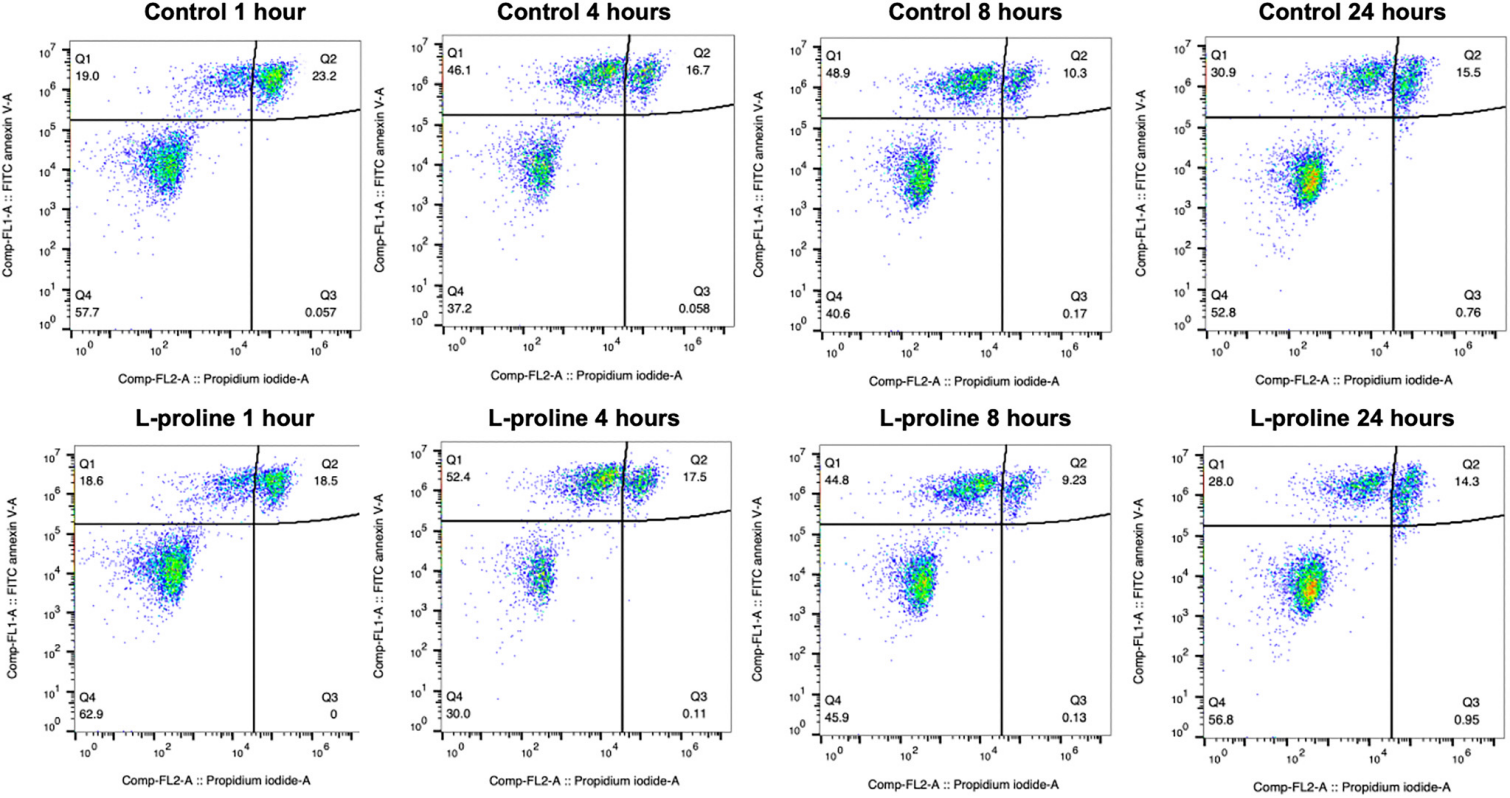
Flow cytometry analysis of apoptosis/necrosis of Jurkat cells previously incubated with and without 200 mM l-proline for 24 hours. FITC+ = early apoptosis FITC+ PI+ = late apoptosis or necrosis. Cells were incubated for 24 hours with fresh media containing 200 mM of l-proline, or no additive. After incubation, cells were counted, concentration readjusted to 10 × 10^6^ cells per mL, and resuspended in fresh media. 5% DMSO was added, cells were cryopreserved and after spending at least 24 hours at −80 °C, cells were thawed.

One indicated mechanism of l-proline's cryoprotective effects is the inhibition of apoptosis by ROS scavenging.^34^ To investigate the effect of l-proline on post-thaw apoptosis we used a FITC annexin V apoptosis assay, in which cells were incubated with fluorescein isothiocyanate (FITC) labelled annexin V (FA), and propidium iodide (PI). Annexin V binds to phosphatidylserine, which is only present on the cell surface during apoptosis. PI is a fluorescent DNA binding dye, which is only able to enter the cells during late apoptosis or necrosis when the cell surface membrane is disrupted. Therefore, cells stained with neither FA nor PI are alive, cells stained with only FA are in early apoptosis, and cells stained with both FA and PI are in late apoptosis or necrosis. These changes must be monitored over a 24-hour period because there is a window of time in which early apoptosis can be detected. If measured too soon, apoptosis will not have started in all the cells destined for it, if measured too late, then the cells will have transitioned to late apoptosis which is indistinguishable from necrosis because the cell membrane becomes permeable to PI in both causes. Jurkat cells were prepared at the 1 × 10^6^ cells per mL, incubated with 200 mM of l-proline and cryopreserved. Cells were stained and florescence analysed by flow cytometry at 1, 4, 8 and 24 hours post-thaw and shown in Table 1 and Fig. 6. Across all conditions there was no clear difference in apoptosis/necrosis profile supporting that another mechanism(s) of action is responsible, linked to the depressed growth rates observed above. A previous whole-cell proteomics analysis of A549 cells did not reveal any specific mechanism of action,^40^ supporting a broad response which decrease cell proliferation preparing cells for the cold stress.

**Table 1:** Results of flow cytometry showing the apoptosis/necrosis profile of Jurkat cells previously incubated with and without 200 mM l-proline for 24 hours. Early = early apoptosis, late = late apoptosis

	1 hour	4 hours	8 hours	24 hours
Control	Live: 58%	Live: 37%	Live: 41%	Live: 53%
	Early: 19%	Early: 46%	Early: 49%	Early: 31%
	Late or necrosis: 23%	Late or necrosis: 17%	Late or necrosis: 10%	Late or necrosis: 16%
l-Proline	Live: 63%	Live: 30%	Live: 46%	Live: 53%
	Early: 19%	Early: 52%	Early: 45%	Early: 31%
	Late or necrosis: 19%	Late or necrosis: 18%	Late or necrosis: 9%	Late or necrosis: 16%

## Conclusions

Here we investigated the impact of l-proline on the cryopreservation of Jurkat cells, which are a useful model for CAR T-cell therapies, showing that l-proline can increase post-thaw cell yields when a 24 hour pre-conditioning period is applied. Crucially no changes are made to the final cryopreservation formulation or process, making this easy to deploy. Our hypothesis was that the previously reported cryoprotective effect of l-proline on cell lines, and on organisms, could be applied to T-cells which are emerging as advanced cell-based therapies that are delivered to patients cryopreserved. We demonstrate that incubation of 200 mM l-proline with Jurkat cells leads to a suppression in their growth rate, but without reducing the cell viability nor their metabolic activity. Cells treated in this manner showed increases in their post-thaw yield with lower cell-densities (0.5 × 10^6^ cells per mL), showing greater increases compared to higher density (1 × 10^6^ cells per mL). This can be explained by the fact that at higher cell densities, Jurkat proliferation rate is already suppressed, and gives high post-thaw yields compared to low density. Hence the l-proline incubation at low density induces the same effect as higher densities. d-Proline was found to have no benefit, decreasing cell yield, supporting a biochemical rather than biophysical mode of action, potentially linked to the inability of proline transporters to tolerate the d-isomer. l-Alanine was also shown to improve cell yield in some conditions, and to also suppress growth rate, suggesting several amino acids can exert a cryoprotective effect in this pre-incubation method, although previous studies on other cells suggest alanine is less effective in general.^40^ A flow cytometry study showed no impact on post-thaw apoptosis upon proline pre-conditioning, again supporting that growth rate suppression is the primary mode of action. Overall these results demonstrate that the pre-conditioning of Jurkat cells with l-proline (and potentially other amino acids) is a viable route to increase post-thaw yields without making any changes to established cryopreservation methods or formulations. These results may aid in developing new methods to increase T-cell yield following cryopreservation which would be of use for emerging cell-based therapies.

## Conflicts of interest

There is no conflict of interest to declare.

## Supplementary Material
